# Direct treatment of interaction between laser-field and electrons for simulating laser processing of metals

**DOI:** 10.1038/s41598-021-94036-4

**Published:** 2021-07-16

**Authors:** Yoshiyuki Miyamoto

**Affiliations:** Research Center for Computational Design of Advanced Functional Materials, National Institute of Advanced Industrial Science and Technology (AIST), Central 2, 1-1-1 Umezono, Tsukuba, Ibaraki 305-8568 Japan

**Keywords:** Lasers, LEDs and light sources, Theory and computation

## Abstract

Laser ablation is often simulated by the two-temperature model in which electrons are assumed to be thermalized by laser irradiation, while an explicit representation of interaction between laser-field and electrons is challenging but beneficial as being free from any adjustable parameters. Here, an ab initio method based on the time-dependent density functional theory (TDDFT) in which electron-ion dynamics under a laser field are numerically simulated is examined as a tool for simulating femtosecond laser processing of metals. Laser-induced volume expansion in surface normal directions of Cu(111) and Ni(111) surfaces are simulated by using repeating slab models. The amount of simulated volume expansion is compared between Cu(111) and Ni(111) slabs for the same laser pulse conditions, and the Ni slab is found to expand more than the Cu slab despite the smaller thermal expansion coefficient of Ni compared with Cu. The analyzed electronic excitation and lattice motion were compared to those in the two-temperature model. The threshold fluence to release surface Cu atom deduced from current TDDFT approach is found to be comparable to those of Cu ablation reported experimentally.

The development of high power lasers^1^ has enabled laser processing of materials by using pulse laser^2–9^. The results of processing, such as melting/drilling depths and sizes of created holes/craters, are functions of laser parameters such as wavelength, power (fluence), and pulse width. The processing also depends on whether the material is a metal, semiconductor, or insulator. When the pulse width is in the order of picosecond, the ablation process is thermal^3^. Meanwhile, the femtosecond laser processing is claimed to be non-thermal^2,9^, there is a need to understand laser processing beyond conventional thermodynamics.

The two-temperature model for electronic and lattice systems^10–20^ is widely used to explain the following laser processing scenario: First, laser-excited electrons relax to thermal equilibrium by electron-electron interactions thereby increasing the electron temperature, and then the lattice temperature is increased by electron-lattice interactions. In the two-temperature model, laser-induced excitation and subsequent lattice dynamics are simulated by an equation for heat transport from electrons to the lattice which uses parameters such as the heat capacities of electrons and the lattice as well as an electron-lattice coupling constant^13,17,20^. This model has been applied to laser ablation of Cu by combining experimental and theoretical approaches^21^. The concept of electron temperature has also been introduced into ab initio calculations^11,12^ used to study nonlinear optical properties^22^, phase changes^23^, and ablation^24^ of transition metals under irradiation with femtosecond laser.

Although, the two-temperature model works practically well, some properties like as polarization dependence of ablation speed^8^ cannot be explained. The present study aims to simulate femtosecond laser processing of metals deductively from ab initio simulation starting with an explicit representation of interaction between laser-field and electrons and subsequent molecular dynamics instead of employing electron temperature. Although there still remains a gap between atomic-scale phenomena and macroscopic-scale phenomena, the first step toward simulating femtosecond laser processing is to monitor the volume expansion and release of surface atoms of metals by using conventional repeating slab models representing metal surfaces. This work presents computational schemes for this step based on the density functional theory (DFT)^25,26^ and the time-dependent density functional theory (TDDFT)^27^ coupled with molecular dynamics (MD). This is referred to as TDDFT-MD simulation in the rest of this manuscript.

Laser-induced volume expansion and release of surface atoms of a Cu(111) surface is then studied in accordance with industrial demands. Moreover, Ni(111) surfaces are also simulated for comparison with the Cu(111) surface and are found to exhibit larger volume expansion. This may be surprising since the thermal expansion coefficient of Ni is smaller than that of Cu. Upon laser excitation, the electronic system is found to be in a non-equilibrium state, and meanwhile that the lattice motion has already started in the corresponding time domain. The possible reasons of lattice motion before thermalization of electronic system in present calculations are discussed. The estimated threshold for releasing surface atoms from Cu(111) surface is then compared with experimentally reported values of Cu ablation. All computational details are presented in the **Methods** section of this manuscript.

## Results and discussions

### Comparison between Cu and Ni slabs

**Figure 1:**
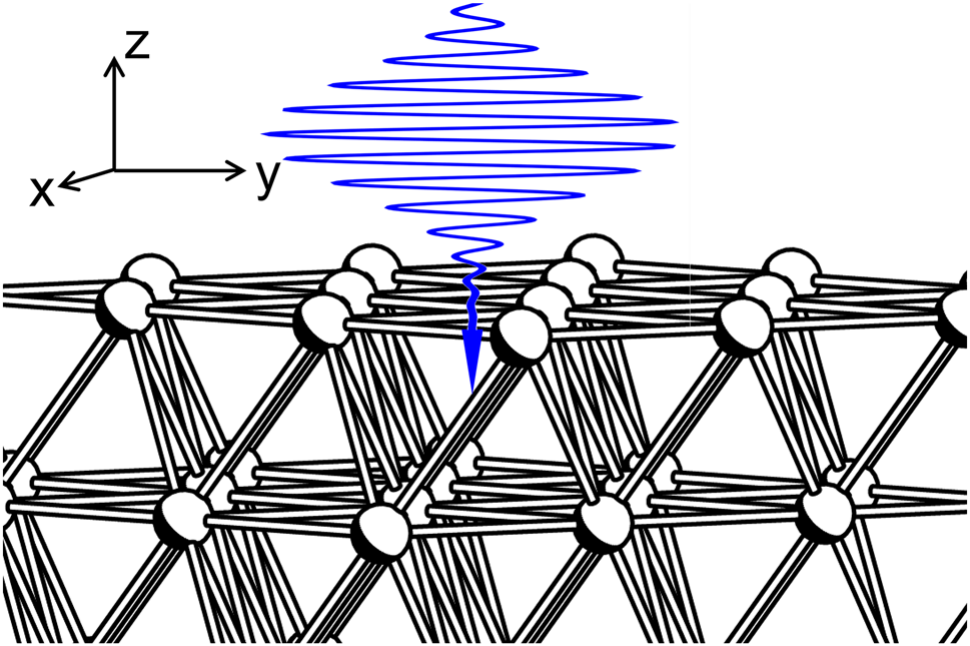
Slab model of a Cu(111) surface. The surface normal direction is along the *z* axis, the (111) crystallographic direction. Wavy line (blue) schematically denotes the incident laser with a polarization direction along the *y* axis, which is along a diagonal of the hexagonal cell of the Cu(111) surface, the $(-1,2,-1)$ crystallographic direction.

**Figure 2:**
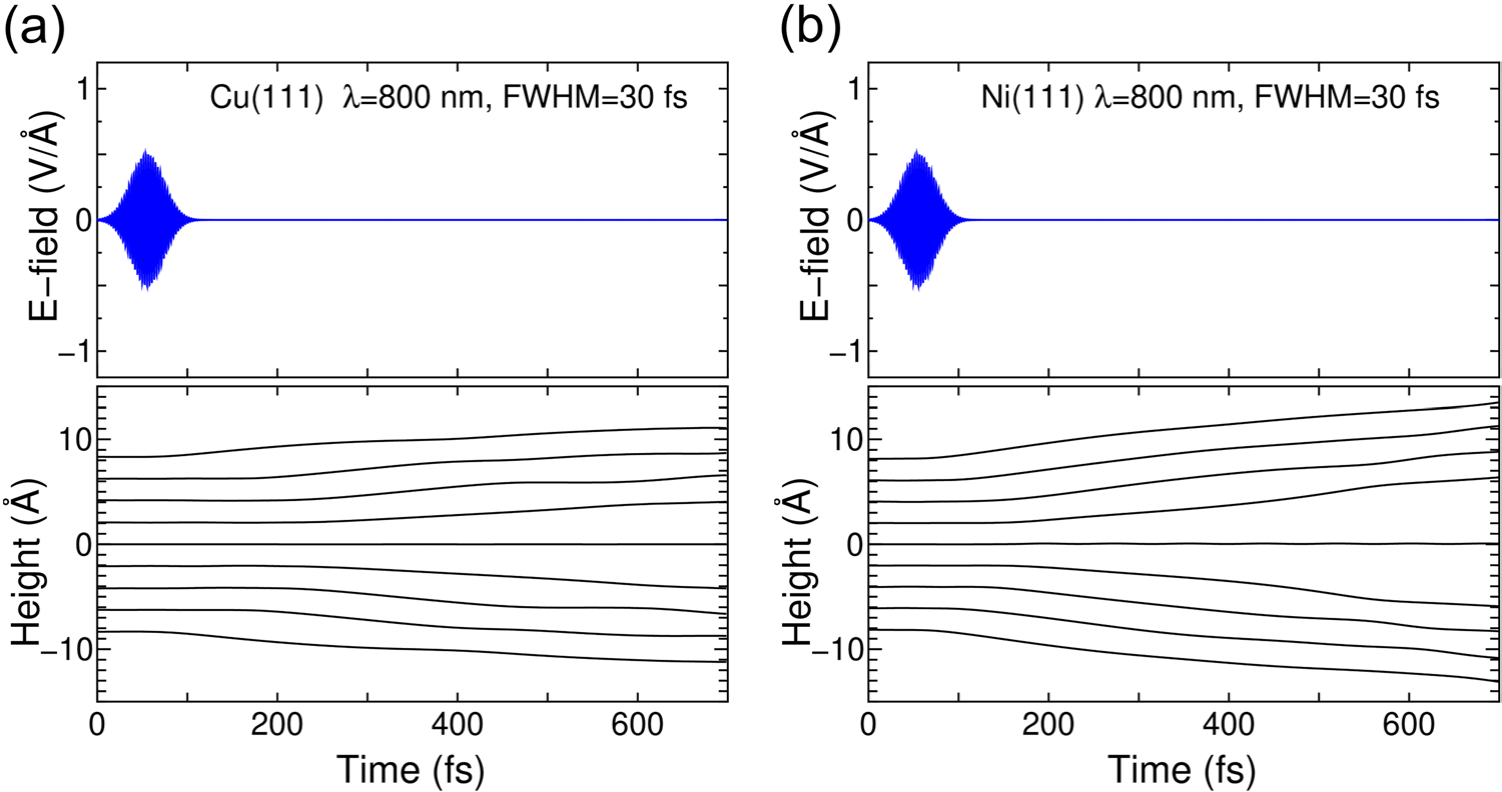
(**a**) TDDFT-MD simulation of a Cu slab upon a laser shot with wavelength of 800 nm, FWHM of 30 fs, and fluence of 0.1 J/cm$^2$. The upper panel shows the time variation of the laser field polarized along the *y* axis of Fig. 1. The lower panel shows the dynamics of atomic height of each layer along the *z* axis of Fig. 1. (**b**) Same as (**a**) but for a Ni slab.

The Cu and Ni slabs are compared by using a 9 atomic-layer model. Figure 1 show a schematic of present condition. The laser incident direction is parallel to *z* axis which is normal to the (111) surface. While the laser field is polarized in *y* direction, which is along with diagonal direction of the hexagonal cell of the (111) surface. Figure 2 shows the results of TDDFT-MD simulations with a laser shot with wavelength of 800 nm, FWHM of 30 fs, and fluence of 0.1 J/cm$^2$.

The assumed slab thickness ($\sim$1.6 nm) is much below typical penetration depth of the laser beam^8^. The results show a larger volume expansion for the Ni(111) slab than for the Cu(111) slab. The larger volume expansion of Ni may be surprising because it has a lower thermal expansion coefficient than that of Cu, but this is consistent with experimental reports of higher infrared absorption by Ni^28^, which is conducive to gaining energy from the laser pulse. The higher infrared absorption can be understood from the difference in band structures between Cu and Ni, which are shown in Sec. S.I in supplementary materials of this manuscript as being consistent with previous calculations^29,30^. The difference originates from the difference in the number of electrons occupying 3*d* orbitals between Cu and Ni. The TDDFT-MD simulation shown in Fig. 2a is continued up to 820 fs and shows continuous volume expansion beyond 135% of the original volume. This indicates that the fluence of 0.1 J/cm$^2$ should be above threshold for releasing atoms in surface regions of both Cu and Ni with FWHM of 30 fs. The specific value of the threshold will be discussed again later with wider FWHM.

To address a possibility of electron emission throughout the TDDFT-MD simulation shown in Fig. 2a,b, the charge redistribution along with surface normal direction (*z* axis in Fig. 1) is checked and no significant emission into the vacuum is found. The details are presented in Sec. S.II of the supplementary materials. It is therefore concluded that the Coulomb explosion cannot cause significant volume expansion. An interesting fact is that all layers except the central ones move together for both Cu and Ni. This peculiar dynamics suggests that the applied fluence of 0.1 J/cm$^2$ with FWHM=30 fs is strong enough to give spatially uniform force field to whole layers except the central one. The exception is due to odd number of total layers of the employed slab model that cancels the force on the central layers. The used slab thickness of 9-layer may not be enough to monitor behavior of real sample in deeper region with this laser condition. The dynamics will differ with increased FWHM and the fluence, as will be discussed later.

### Analysis of laser-excited state of Cu slab model

The laser-excited state of the Cu slab model is analyzed. A thicker slab containing 15 atomic layers is used while keeping the laser conditions as FWHM = 30 fs, fluence = 0.1 J/cm$^2$. The usage of the thicker slab is to make the calculated density of states (DOS) understandable in terms of the electronic structure of bulk Cu.

**Figure 3:**
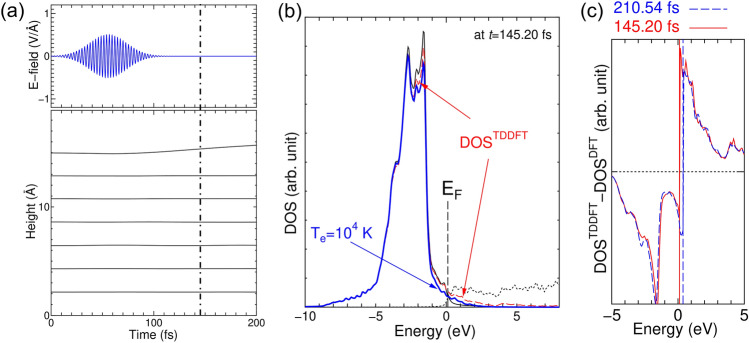
(**a**) TDDFT-MD simulation of Cu(111) 15 atomic-layer slab upon a laser shot with wavelength of 800 nm, FWHM of 30 fs, and fluence of 0.1 J/cm$^2$. The upper panel shows the laser field, while the lower panel shows the height of each layer as a function of time. (Only the upper half of the slab is shown.) (**b**) Thick dash (red) curve is DOS^TDDFT^ at *t* = 145.20 fs (corresponding to the dot-dashed vertical line in (**a**)). The DOS^DFT^ with atomic coordinates at the snapshot (*t* = 145.20 fs) of the TDDFT-MD simulation is shown by solid (dotted) curve below (above) the E_F_. The thick solid (blue) curve shows the DOS^DFT^ weighted by FDD with electronic temperature of 10^4^ K. (**c**) Time evolution of the DOS^TDDFT^-DOSDOS^DFT^ with the same atomic coordinates. Solid (red) line is at *t* = 145.20 fs, while the dash (blue) line is at *t* = 210.54 fs. Note that the vertical lines are location of the E_F_ determined by the DFT calculation at each snapshot and that the dotted horizontal line is zero value of DOS^TDDFT^-DOS^DFT^.

Figure 3a shows a TDDFT-MD simulation under the laser shot. The lattice expansion starts around 110 fs, at which point the laser field decays. The simulation is up to 200 fs and the dynamics of each layer is very similar to those of 9-layer slab as displayed in Fig. 2a. Meanwhile, Fig. 3b shows the DOS obtained by TDDFT-MD simulation (DOS$^{{\rm TDDFT}}$) at *t* = 145.20 fs by projecting the $\psi ^{TDDFT}_{\mathrm{n,\mathbf{k}}}(\mathbf{r},t)$ onto the Kohn-Sham orbitals $\psi ^{DFT}_{\mathrm{n, \mathbf{k}}}(\mathbf{r})$ obtained by the static DFT simulation with atomic coordinates the same as at the snapshot (*t*= 145.20 fs) of the TDDFT-MD simulation. (For numerical details, see Sec. S.III of the supplementary materials.) The high DOS peaks below the Fermi Level (E$_{\mathrm{F}}$) (0 eV) in Fig. 3b match the flat-dispersion region of the band structure of bulk Cu, see red arrows indicated in Fig. S.1a in the supplementary materials of this manuscript. The lower (higher) DOS$^{{\rm TDDFT}}$ compared with the DOS by DFT simulation (DOS$^{{\rm DFT}}$) in the valence (conduction) band region denotes the creation of holes in valence bands (creation of electrons in conduction bands).

Currently obtained DOS$^{{\rm TDDFT}}$ was compared to the Fermi-Dirac distribution (FDD) with electronic temperature of 10$^4$ K, as shown in blue curve in Fig. 3b. The DOS$^{{\rm TDDFT}}$ shows higher DOS in valence region (underestimated hole density) while shows higher DOS in conduction region (overestimated electron density). In order to obtain reasonable fit to the FDD, both of hole and electron densities should be simultaneously underestimated (otherwise overestimated). This means neither increase nor decrease of the electron temperature can fit the FDD to the DOS$^{{\rm TDDFT}}$. This deviation from the FDD after the excitation has been discussed in Ref.^20,31^], in which the electronic system was interpreted not to be equilibrated in the early time domain with the lattice remaining cool. However, the present TDDFT-MD simulation indicates that the lattice dynamics have already started even in the early time domain with non-equilibrium conditions in the electronic system.

Further interest is how $DOS^{{\rm TDDFT}}$ evolves with respect to time. Figure 3c shows the ${\mathrm{DOS}}^{{\rm TDDFT}}$ subtracted by ${\mathrm{DOS}}^{{\rm DFT}}$ of the snapshot of the TDDFT-MD simulation at *t* = 145.20 fs and *t* = 210.54 fs. The negative value of the DOS means creation of holes below ${\mathrm{E}}_{\mathrm{F}}$ while the positive value of the DOS means creation of excited electrons above ${\mathrm{E}}_{\mathrm{F}}$. Within this time variation, relaxation of hole and electron toward the ${\mathrm{E}}_{\mathrm{F}}$ was not significant. On the other hand, experimentally analyzed time-constant of electron-thermalization was in the order of few 100 fs^32,33^ which is comparable time-constant of current simulation. Absence of electron-thermalization in current TDDFT-MD simulation can be attributed to small size of currently used slab model that may limit the interaction channels among the time-dependent Kohn-Sham wavefunctions, or attributed to intrinsic problem of the TDDFT lacking the memory effect in the exchange-correlation potential as was discussed in Ref.^34^]. Furthermore, first-principles electron dynamics in bulk Cu suggests different electron temperatures for 3*d* orbital and 4*s*, 4*p* orbitlals^35^. As deduced from these facts, it is concluded that conversion of the data obtained from present ab initio results to parameters for the “two temperature model” is not straightforward.

### Comparative dynamics of Cu slabs under fluences of 0.1 J/cm^2^ and 0.2 J/cm^2^

**Figure 4:**
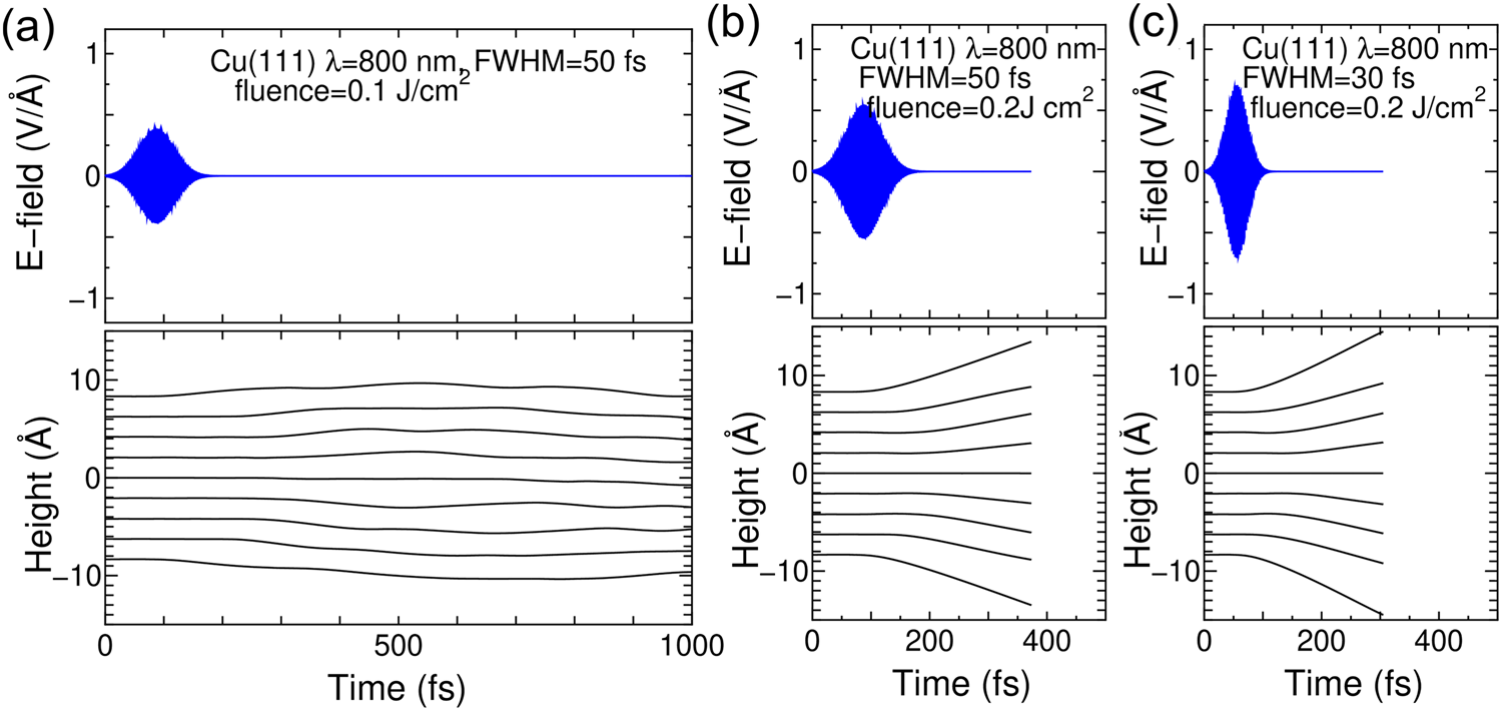
(**a**) TDDFT-MD simulation of a Cu slab upon a laser shot with wavelength of 800 nm, FWHM of 50 fs, and fluence of 0.1 J/cm^2^. The upper panel shows the time variation in the laser field polarized along the *y* axis of Fig. 1. The lower panel shows the dynamics of atomic height of each layer along the *z* axis of Fig. 1. (**b**) Same as (**a**) but with fluence 0.2 J/cm^2^. (**c**) Same as (**b**) but with FWHM=30 fs.

The threshold for releasing surface atoms is examined by increasing the FWHM to 50 fs or by increasing the fluence by 0.2 J/cm^2^. The lattice dynamics with FWHM = 50 fs under fluences of 0.1 J/cm^2^ and 0.2 J/cm^2^ are compared as shown in Fig. 4a and b. For a fluence of 0.2 J/cm^2^, the FWHM=30 fs was also tested (Fig. 4c). The optical wavelength is kept at 800 nm and 9 atomic-layer slab model is used again.

The dynamics under a fluence of 0.1 J/cm^2^ with FWHM=50 fs are monitored up to 1000 fs (1 ps) and exhibited a volume oscillation. When the fluence is increased to 0.2 J/cm^2^, release of surface atoms is seen within a short time constant of less than 400 fs. One can note that the heights of top/bottom atoms change linearly with time suggesting constant velocities and thus no forces. At this moment the inter atom distances are beyond 4 Å with corresponding kinetic energies over 1 eV. These facts warrant release of surface atoms. (Note that further continuation of the simulation gives collision of the emitted ions to opposite surfaces due to the periodic boundary condition, thus the simulation was suspended at this time.) Therefore, with FWHM=50 fs, the threshold fluence for releasing surface atoms should be between 0.1 J/cm^2^ and 0.2 J/cm^2^.

By comparing the results with FWHM=30 fs shown in Fig. 2a and b, all interlayer distances oscillate with fluence of 0.1 J/cm^2^. The origin of volume oscillation in Fig. 4a is inter Cu-atom force superior to laser induced force of expansion, but giving longer oscillation period than typical optical phonon mode of Cu (142 fs for 7 TH$_{\mathrm{Z}}$ frequency). Meanwhile the interlayer distance increases with fluence of 0.2 J/cm^2^ particularly between the surface layers and their neighbors. This particular increase is also the case with fluence 0.2 J/cm$^{2}$ but with FWHM=30 fs, see Fig. 4c. Contrary to the case with fluence of 0.1 J/cm^2^, the atom release occurs mostly in surface few-layer region, thus the thickness of 9-layer slab is plenty good to express the laser induced dynamics with fuence of 0.2 J/cm$^{2}$. The different interlayer dynamics from cases with fluence of 0.1 J/cm$^{2}$ could be attributed to spatial non-uniformity of excited electronic systems with increased fluence.

Note that the value of currently obtained threshold between 0.1 J/cm$^{2}$ and 0.2 J/cm$^{2}$ is comparable to the upper bound of the reported ablation threshold (< 0.18 J/cm$^{2}$) with FWHM of 70 fs and the same wavelength^36^, and is comparable to a recent experimental value of 0.137 J/cm$^{2}$ with FWHM of 100 fs and the same wavelength^21^. Even though the present simulation does not precisely express complex process of ablation, and the assumed laser conditions (pulse width, number of laser shots, and assumption of atomically flat surface) are different from those in experimental reports, the consistency in the order of the threshold value is a promising sign for future realistic simulation using larger models. Potential reason of consistence in thresholds is that experimental values of ablation^21,36^ were determined by analysis of depth of heating propagation and depth of ablation crater which should mostly be determined by vertical momentum of lattice being monitored in the current simulations.

## Concluding remarks

In conclusion, the TDDFT-MD simulation demonstrated the ability to provide microscopic results showing volume expansion depending on the laser parameters and materials. From current TDDFT-MD simulation, faster volume expantion of Ni than Cu is observed. Although the electron temperature could not be extracted, present calculations show threshold fluence of atoms leaving from Cu surface with FWHM=50 fs being in the order of previous experimentally reported values for ablation. It should also be noted that similar simulation with surface normal polarization of laser field with wavelength 800 nm, FWHM = 50 fs was tested and gave no significant lattice motion even with higher fluence of 1 J/cm$^{2}$, thus the results depend on the polarization.

Given that the present simulation runs on an atomic scale, further interpretation of the present results is necessary in order to perform larger scale simulation considering factors such as laser beam size and surface irregularity. One way to use current results for large scale simulation could be applying fluence-dependent ablation data to spatially dependent dynamics in accordance with the realistic intensity profile of the laser spots.

## Methods

The simulations curried here is based on the DFT^25,26^. The electron-ion dynamics using real-time TDDFT scheme^27^ are calculated under the presence of a laser field coupled with a classical MD simulation within the Ehrenfest approximation^37^. The Perdew-Zunger functional for the local density approximation (LDA)^38^ is used to represent the exchange-correlation energy. Interactions between ions and valence electrons are expressed by using norm-conserving pseudopotentials^39^ with separable forms^40^. For Cu and Ni fcc structures, the LDA functional gives agreement in the lattice constant within +1% when used with norm-conserving pseudopotentials made in accordance with the reported recipe^41,42^. Because the agreement is better than that of the generalized gradient approximation with the PBE functional^43^, LDA is used for all calculations in this work.

Within a scheme of TDDFT, the electron dynamics under laser field is expressed by the time-dependent Kohn-Sham equation^27^ with the presence of a uniform optical field ${\mathbf{E}}_{ext}(t)$^44,45^ (in length gauge):

1 $$\begin{aligned} i\frac{\partial }{\partial t}\psi ^{TDDFT}_{\mathrm{n,\mathbf{k}}}(\mathbf{r},t) =\left\{ H^{KS}(\mathbf{r},t)+{\mathbf{r}}\cdot {\mathbf{E}}_{ext}(t)\right\} \psi ^{TDDFT}_{\mathrm{n,\mathbf{k}}}(\mathbf{r},t). \end{aligned}$$

In this work, all equations are expressed in the atomic unit, in which mass and charge of single electron as well as the reduced Planck constant are set as unity. $\psi ^{TDDFT}_{\mathrm{n,\mathbf{k}}}(\mathbf{r},t)$ is the time-dependent Kohn-Sham orbital with band ${\mathrm{n}}$ and wave vector ${\mathrm{\mathbf{k}}}$, and $H^{KS}(\mathbf{r},t)$ is the Kohn-Sham Hamiltonian. The ${\mathbf{E}}_{ext}(t)$ is set spatially uniform since the optical wavelength of 800 nm applied in this work is much longer than the model size (long-wavelength approximation). In this long-wavelength approximation, the propagation of laser beam was not included in the atomic scale simulation since the entire region of the unitcell uniformly feels the laser field. In consideration of the surface normal incidence of the laser pulse, the polarization vector of light is set as parallel to the (111) surface. Therefore, the dipole ${\mathbf{r}}\cdot {\mathbf{E}}_{ext}(t)$ does not suit the periodic boundary conditions in the surface parallel directions. As discussed in a review paper^46^, one way to solve this problem is to change Eq. (1) from length gauge to velocity gauge using the vector potential ${\mathbf{A}}_{ext}(t)=\int ^t{\mathbf{E}}_{ext}(t')dt'$. Then a new wavefunction

2 $$\begin{aligned} {\tilde{\psi }}^{TDDFT}_{\mathrm{n,\mathbf{k}}}(\mathbf{r},t)=e^{i{\mathbf{r}}\cdot {\mathbf{A}}_{ext}(t)}\psi ^{TDDFT}_{\mathrm{n,\mathbf{k}}}(\mathbf{r},t). \end{aligned}$$

is introduced. The new equation in velocity gauge obtained by substituting Eq. (2) into Eq. (1) is

3 $$\begin{aligned} i\frac{\partial }{\partial t}{\tilde{\psi }}^{TDDFT}_{\mathrm{n,\mathbf{k}}}(\mathbf{r},t) =e^{i{\mathbf{r}}\cdot {\mathbf{A}}_{ext}(t)}H^{KS}(\mathbf{r},t)e^{-i{\mathbf{r}}\cdot {\mathbf{A}}_{ext}(t)}{\tilde{\psi }}^{TDDFT}_{\mathrm{n,\mathbf{k}}}(\mathbf{r},t).~~ \end{aligned}$$

Since the norm of the wavefunctions is preserved, the Eq. (3) gives the same dynamics of charge density $\rho (\mathbf{r},t)$ as the length gauge. Hereafter, the new wavefunctions ${\tilde{\psi }}^{TDDFT}_{\mathrm{n,k}}(\mathbf{r},t)$ is replaced with $\psi ^{TDDFT}_{\mathrm{n,k}}(\mathbf{r},t)$.

Generally, the Kohn-Sham Hamiltonian $H^{KS}(\mathbf{r},t)$ is written as

4 $$\begin{aligned} H^{KS}(\mathbf{r},t)=-\frac{1}{2}\nabla ^2+V_{nonloc}(\mathbf{r},{\mathbf{r}}',t)+V_{loc}(\mathbf{r},t), \end{aligned}$$

where the first term on the right-hand side is the kinetic energy operator, the second term is all non-local parts of the pseudopotentials, and the last term is the local part of the pseudopotentials in addition to the Hartree and exchange-correlation terms in DFT. Therefore, the time-dependent equation with the velocity gauge (3) is derived as^47^

5 $$\begin{aligned} i\frac{\partial }{\partial t}\psi ^{TDDFT}_{\mathrm{n,\mathbf{k}}}(\mathbf{r},t) =\left \{\frac{1}{2}\left(\frac{1}{i}\nabla -{\mathbf{A}}_{ext}(t)\right)^2 +V_{loc}(\mathbf{r}.t)\right \}\psi ^{TDDFT}_{\mathrm{n,\mathbf{k}}}(\mathbf{r},t)+e^{i{\mathbf{r}}\cdot {\mathbf{A}}_{ext}(t)}\int V_{nonloc}(\mathbf{r},{\mathbf{r}}',t)e^{-i{\mathbf{r}}'\cdot {\mathbf{A}}_{ext}(t)}\psi ^{TDDFT}_{\mathrm{n,\mathbf{k}}}(\mathbf{r}',t)d{\mathbf{r}}'. \end{aligned}$$

By using the plane-wave basis set to express $\psi ^{TDDFT}_{\mathrm{n,\mathbf{k}}}(\mathbf{r},t)$, the computation of the first and the last terms on the right-hand side of Eq. (5) is straightforward. One can shift the wave vector of a plane wave from ${\mathbf{G}}+{\mathbf{k}}$ to ${\mathbf{G}}+{\mathbf{k}}-{\mathbf{A}}_{ext}(t)$, where ${\mathbf{G}}$ is the reciprocal vectors of the unit cell and ${\mathbf{k}}$ is the vectors at *k* points within the first Brillouin zone. This treatment differs from using localized basis set^47^. Time integration of Eq. (5) for the real-time TDDFT simulation is performed by using a fourth-order split-operator scheme^48,49^ with a time step $\Delta t$ of 0.03 a.u. (7.26$\times$10$^{-4}$ fs).

Note that the shifted wave vector ${\mathbf{G}}+{\mathbf{k}}-{\mathbf{A}}_{ext}(t)$ is also used to compute the contribution of non-local pseudopotentials to the Hellman-Feynman forces in reciprocal space^50^ expressed as

6 $$\begin{aligned} -\sum _{\mathrm{n,\mathbf{k}}}\int \int \psi ^{TDDFT}_{\mathrm{n,\mathbf{k}}}(\mathbf{r},t)^*e^{i{\mathbf{r}}\cdot {\mathbf{A}}_{ext}(t)}\frac{\partial V_{nonloc}(\mathbf{r},{\mathbf{r}}',t)}{\partial {\mathbf{R}}_I(t)}e^{-i{\mathbf{r}}'\cdot {\mathbf{A}}_{ext}(t)}\psi ^{TDDFT}_{\mathrm{n},\mathbf{k}}(\mathbf{r}',t)d\mathbf{r}'d\mathbf{r},~~ \end{aligned}$$

where ${\mathbf{R}}_I(t)$ is the coordinate of the *I*th ion. In addition to this term, including Coulomb interaction between ions and the total charge of electrons makes the Hellman–Feynman forces contributing to the electron-lattice interaction. Since the total charge and Kohn-Sham orbitals can be modified by irradiation with laser pulses, the laser modulates the Hellman–Feynman forces. In this way, laser-induced electron-phonon collision was approximately computed. Yet, the momentum exchange upon electron-phonon collision is restricted under given size of the unitcell.

A plane-wave basis set with a cutoff kinetic energy of 62 Ry is used to express $\psi ^{TDDFT}_{\mathrm{n},\mathbf{k}}(\mathbf{r},t)$ and charge density. This cutoff kinetic energy is enough to reproduce the lattice constant of fcc Cu and Ni within an error of 1 % and their bulk moduli with $\sim$ 10 % errors. The charge density is used to express Hartree and exchange correlation potentials in $H^{KS}(\mathbf{r},t)$. A self-consistent relationship is kept between time-evolving $\psi ^{TDDFT}_{\mathrm{n},\mathbf{k}}(\mathbf{r},t)$ and $H^{KS}(\mathbf{r},t)$^51,52^, so electron-electron interaction within DFT level is included. The total energy and forces are computed by the momentum-space formalism^50^. The computed forces are used for molecular dynamics (MD) calculations performed simultaneously with the real-time TDDFT simulation. The TDDFT-MD simulations are performed using fpseid code^51,52^. Simulations using velocity gauge and using length gauge are expected to agree in the case of an isolated system, as discussed in the review paper^46^. Agreement was checked in the case of a hydrogen fluoride molecule (see Sec. S.IV in the supplementary materials of this manuscript).

The time dependence of the optical field is assumed to be

7 $$\begin{aligned} {\mathbf{E}}_{ext}(t)={\mathbf{E}}_{0}e^{-\alpha (\frac{t-t_0}{\tau })^2}\sin {\omega t}, \end{aligned}$$

with the dimensionless parameter $\alpha$=1/2ln2. The frequency $\omega$ is set to the corresponding optical wavelength of 800 nm. The parameter $\tau$ determines the pulse width which is set to 15 fs (25 fs) combined with parameter $t_0$ set to 55.5 fs (87.5 fs) corresponding to a full width at half-maximum (FWHM) of 30 fs (50 fs). $\mid {\mathbf{E}}_0\mid$ gives the maximum laser power and is set to match the laser fluence with given FWHM values (see Table 1).

**Table 1:** Values of $\mid {\mathbf{E}}_0\mid$ combined with fluence (J/cm$^{2}$) and FWHM (fs) used in the present work.

Fluence (J/cm$^{2}$)	FWHM (fs)	$\mid {\mathbf{E}}_0\mid$ (V/Å)
0.1	30	0.50119
0.1	50	0.38822
0.2	50	0.54903

Figure 1 shows the slab model of Cu(111). The (111) surface has hexagonal cells extending in the *xy* directions in which the $1\times 1$ period is taken. Since current work focuses on ion dynamics merely in surface normal direction to examine volume expansion and atom release, the minimum period in the surface lateral direction was employed. Thus the 9-layer slab model contains 9 atoms per unit cell. Volume expansion and atom release are intensively studied by using a 9 atomic-layer slab model, in which a vacuum region of 14.3 Å is taken for the periodic boundary condition in the *z* direction. This spacing in the *z* direction is needed in order to simulate laser-induced volume expansion and atom release. A 15 atomic-layer slab model (containing 15 atoms per unit cell) is also used with a 7.85 Å vacuum region for analyzing only electronic excitation. The polarization of laser field is set as the *y* direction, which is a diagonal of the hexagonal cell of the (111) surface. Thirty irreducible ${\mathbf{k}}$ vectors are used as sampling points in the momentum space to construct the time-dependent charge density $\rho (\mathbf{r},t)$ as a sum of norms of all occupied time-dependent Kohn-Sham orbitals $\psi ^{TDDFT}_{\mathrm{n},\mathbf{k}}(\mathbf{r},t)$. The number of ${\mathbf{k}}$ vectors are enough to reproduce the density of states deduced from the band structure of both Cu and Ni. (See Sec. S.I in the supplementary materials of this manuscript).

Before performing the TDDFT-MD simulation, geometry optimization under the electronic ground state is performed within the DFT. 110 bands (118 bands) are used for 9-layer (15-layer) slab model to solve the Kohn-Sham Eq.^26^. The E$_{\mathrm{F}}$ is determined by using the tetrahedron technique^53^, which determines the electron occupation numbers for Kohn-Sham orbitals at each ${\mathbf{k}}$ vector. The occupation numbers are then kept constant throughout the TDDFT-MD simulation under laser irradiation. To perform the TDDFT-MD calculation, the number of bands are reduced to 56 bands (96 bands) for 9-layer (15-layer) slab model. The reduction is able since the TDDFT calculation needs to treat only occupied bands to generate the time-dependent charge density.

## Supplementary Information

Supplementary Information.

## Data Availability

Te data that support the findings of this study are available from the corresponding author upon reasonable request.
