# Supplementary material for: Direct treatment of interaction between laser-field and electrons for simulating laser processing of metals
Source: Sci Rep. 2021 Jul 16;11:14626. doi: 10.1038/s41598-021-94036-4 (PMC8285478; doi:10.1038/s41598-021-94036-4)
Supplement: Supplementary file 1 — Supplementary Information. [file 41598_2021_94036_MOESM1_ESM.pdf]

Supplementary materials for "Direct Treatment of Interaction between Laser-Field and Electrons for Simulating Laser Processing of Metals"

Yoshiyuki Miyamoto[1,*]

[1]Research Center for Computational Design of Advanced Functional Materials, National Institute of Advanced Industrial Science and Technology (AIST), Central 2, 1-1-1 Umezono, Tsukuba, Ibaraki 305-8568, Japan
[*]yoshi-miyamoto@aist.go.jp

ABSTRACT

The supplementary materials show followings; In Sec. S.I electronic structures of Cu and Ni with use of currently employed pseudopotentials within the local density approximation are shown. In Sec. S.II, redistribution of charge density of Cu(111) and Ni(111) surface upon laser pulse irradiation is shown. In Sec. S.III, the formalisms to compute the density of state at a snapshot throughout the time-dependent density functional calculations is presented. In Sec. S.IV precision check of a photo-induced dynamics of an isolated molecule using length and velocity gaues to express interaction between laser field and electrons is demonstrated.

S.I Band structures of Cu and Ni with current schemes

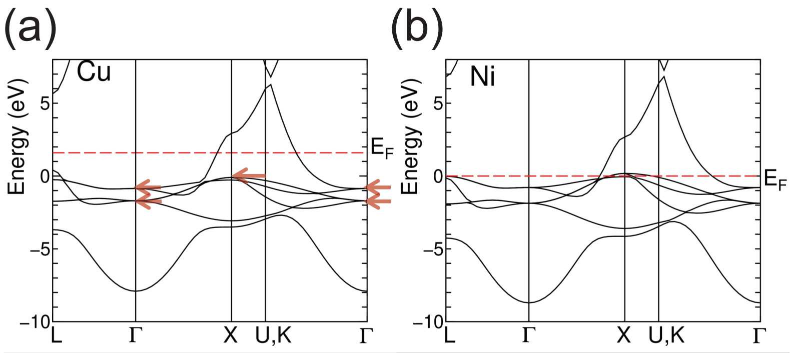

Figure S.1. (Color online) (a) Band structure of the Cu fcc cell. (b) Same as (a) but for Ni. The dash (red) line denotes the location of the E_F. Arrows (red) in (a) denotes DOS peaks below the E_F.

The band structures of the Cu and Ni fcc unit cells are examined by using the generated norm-conserving pseudopotentials and LDA. Calculations are performed using the theoretically determined lattice constant, which is +1% overestimated from the experimental value. Since Cu 3*d* orbitals are fully occupied, only one band crosses the Fermi level (E_F). Meanwhile, the Ni 3*d* orbitals are partially occupied, causing multiple bands to cross the E_F. These band structures are consistent with previous calculations for Cu[1] and Ni.[2] (Note that the calculations in Refs. [1, 2] were performed with schemes of higher precision than current one. Yet, the entire profiles shown in Figs. S.1 (a) and (b) are approximated well thanks to the recipe[3,4] in generating pseudopotentials.) This difference in the electronic structures between Cu and Ni causes the DOS peak of Ni to be much closer to the E_F than that of Cu, and could be the reason for the stronger infrared absorption of Ni.[5] Arrows (red) in Fig. S.1 (a) denote energy positions of DOS peaks as shown in Fig. 3 (b) of the main text.

S.II Charge modulation upon excitation

The charge redistribution throughout the TDDFT-MD simulation was analyzed throughout the TDDFT-MD simulation shown in Figs, 2 (a), (b) of the main text. The charge redistribution along with surface normal direction (z axis in Fig. 1 of the main

text) was checked. Figure S.2 shows a one-dimensional plot of the charge density of Cu and Ni (111) surface along with the z axis, and the plot of difference of charge densities between at t=159.72 fs (t=145.20 fs) and at t=0 fs for Cu (Ni) case. The bottom panels of Figs. S.2 do not show significant emission into the vacuum region, instead show redistribution due to photo-excitation and lattice motion.

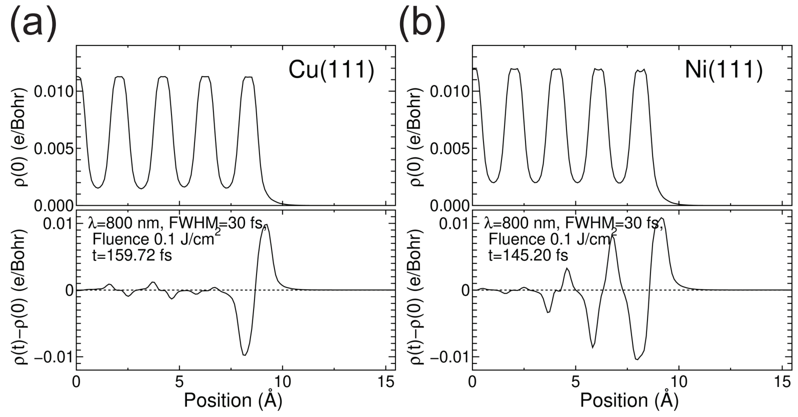

Figure S.2. (Color online) (a) One-dimensional plot of the charge density of Cu(111) slab along with z axis of Fig. 1 of the main text. Upper panel shows the charge density at t=0. Position 0 Å in the lateral axis meets the center of the slab while the right end position meets the middle of the vacuum region. The bottom panel shows charge density of TDDFT-MD simulation at t=159.72 fs subtracted by the charge density of the upper panel. In both panels the charge are averaged in surface parallel directions. (b) Same as (a) but for Ni and the bottom panel is at t=145.20 fs.

S.III DOS calculation at a snapshot of TDDFT-MD simulation

The DOS of TDDFT-MD simulation at t was calculated as

$$DOS^{TDDFT}(\varepsilon,t) = \frac{1}{\pi} Im \sum_{\mathrm{n},\mathbf{k}} \frac{P^{TDDFT}_{\mathrm{n},\mathbf{k}}(t)}{\varepsilon - \varepsilon_{\mathrm{n},\mathbf{k}} + i0^+}, \tag{S.1}$$

where ε is the energy level, $\varepsilon_{\mathrm{n},\mathbf{k}}$ is the eigenvalue of $\psi^{DFT}_{\mathrm{n},\mathbf{k}}(\mathbf{r})$, which corresponds to the Kohn-Sham orbitals obtained by static DFT simulation with the atomic coordinates of a snapshot of TDDFT-MD simulation at t=145.2 fs. The energy broadening factor 0^+ is set to 0.1 eV. The projection $P^{TDDFT}_{\mathrm{n},\mathbf{k}}(t)$ in equation (S.1) is then computed as

$$P^{TDDFT}_{\mathrm{n},\mathbf{k}}(t) = \sum_m f_{m,\mathbf{k}} \mid \int \psi^{TDDFT}_{\mathrm{m},\mathbf{k}}(\mathbf{r},t)^* \psi^{DFT}_{\mathrm{n},\mathbf{k}}(\mathbf{r}) d\mathbf{r} \mid^2, \tag{S.2}$$

where the occupation number $f_{\mathrm{m},\mathbf{k}}$ for $\psi^{TDDFT}_{\mathrm{m},\mathbf{k}}(\mathbf{r},t)$ is fixed independent of t.

S.IV Precision check of TDDFT-MD simulation using length and velocity gauges for a hydrogen fluoride molecule

The computed results of the photo-induced dynamics of an isolated system using length and velocity gauges are expected to be the same. TDDFT-MD simulations of a hydrogen fluoride (HF) molecule are tested under an optical field with wavelength of 800 nm, maximum optical field of 4 V/Å, and FWHM of 10 fs. The direction of polarization is along the HF molecular axis. A periodic box of 5 Å×5 Å×20 Å containing the HF molecule was taken for the periodic boundary conditions with the longest period 20 Å along with HF molecular axis. Nonlocal pseudopotentials are used for the 2s orbital of the fluorine atom, and the plane-wave basis set with a cutoff energy of 62 Ry, as well as the Γ sampling point, is used. A sawtooth-type modulation was introduced for the length gauge by inverting the polarity of $\mathbf{r} \cdot \mathbf{E}_{ext}(t)$ in the vacuum region.

As can be seen in Fig. S.3, the dynamics of H and F atoms obtained by TDDFT-MD simulations in length and velocity gauges are almost identical. The charge modulation at 20 fs is displayed in Fig. S.4. A one-dimensional charge plot taken along the HF axis is shown at 0 fs and 20 fs of simulation time. As highlighted in the insets of Fig. S.4, the charge modulations obtained by TDDFT-MD simulation in length gauge and velocity gauge are almost identical.

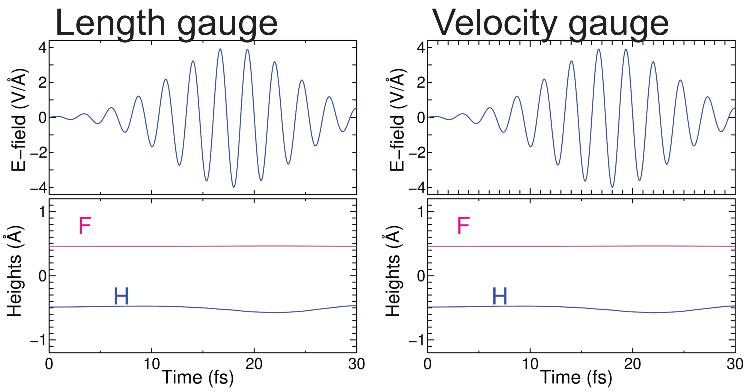

Figure S.3. (Color online) TDDFT-MD simulation of a HF molecule. Upper panels show time evolution of the optical field polarized parallel to the molecular axis. Lower panels show the time evolution of positions of hydrogen (H) and fluorine (F) atoms. The left panels were obtained using length gauge and the right panels were obtained using velocity gauge.

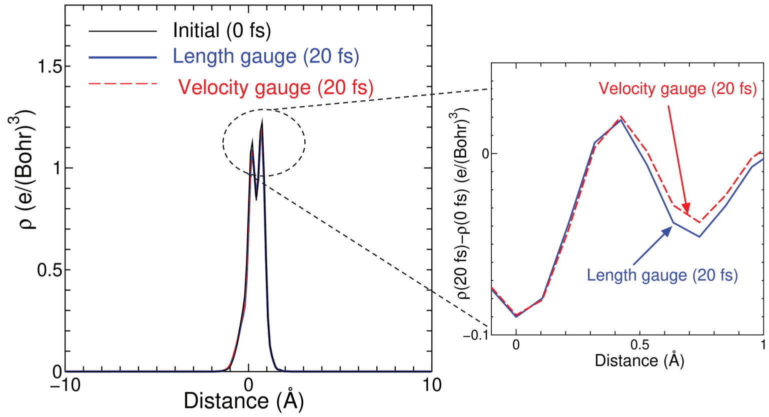

Figure S.4. (Color online) Time evolution of the one-dimensional charge profile along the HF axis. The horizontal axis is positioned corresponding to the vertical axes in Fig. S.3. The plot was taken as t=0 fs and 20 fs. The thin solid (black) line represents the plot at t=0 fs. At t=20 fs, the thick (blue) solid curve is obtained by using the length gauge while the thin dashed (red) line is obtained by the velocity gauge. The inset highlights $\rho(t{=}20\text{ fs})$-$\rho(t{=}0\text{ fs})$ for both gauges at the marked region.

References

1. Gerosa, M., Bottani, C. E., Valentin, C. D., Onida, G. & Pacchioni, G. Accuracy of dielectric-dependent hybrid functionals in the prediction of optoelectronic properties of metal oxide semiconductors: a comprehensive comparison with many-body *GW* and experiments. *J. Phys,: Condens. Matter* **30**, 044003 (2018).
2. Abdallah, L. S. *et al.* Optical conductivity of $Ni_{1-x}Pt_x$ alloys ($0 < x < 0.25$) from 0.76 ev to 6.6 ev. *AIP Advances* **4**, 017102 (2014).
3. Kobayashi, K. Norm-conserving pseudopotential database (NCPS97). *Comput. Mater. Sci.* **14**, 72–76 (1999).
4. Kobayashi, K. A database for norm-conserving pseudopotential (NCPS2K): Application to rare gas atoms. *Mater. Trans.* **42**, 2153–2156 (2001).
5. Biondi, M. A. & Guobadia, A. I. Infrared absorption of aluminum, copper, lead and nickel at 4.2° K. *Phys. Rev.* **166**, 667–673 (1967).
